# Neurodegeneration and Sensorimotor Deficits in the Mouse Model of Traumatic Brain Injury

**DOI:** 10.3390/brainsci8010011

**Published:** 2018-01-06

**Authors:** Saurav Bhowmick, Veera D‘Mello, Nizmi Ponery, P. M. Abdul Muneer

**Affiliations:** Laboratory of CNS Injury and Repair, Neuroscience Institute, JFK Medical Center, 65 James St, Edison, NJ 08820, USA; Sbhowmick@jfkhealth.org (S.B.); veeradmello@gmail.com (V.D.); nizmi.ponery@gmail.com (N.P.)

**Keywords:** traumatic brain injury, fluid percussion injury, neurodegeneration, apoptosis, sensorimotor deficit

## Abstract

Traumatic brain injury (TBI) can result in persistent sensorimotor and cognitive deficits, which occur through a cascade of deleterious pathophysiological events over time. In this study, we investigated the hypothesis that neurodegeneration caused by TBI leads to impairments in sensorimotor function. TBI induces the activation of the caspase-3 enzyme, which triggers cell apoptosis in an in vivo model of fluid percussion injury (FPI). We analyzed caspase-3 mediated apoptosis by terminal deoxynucleotidyl transferase dUTP nick end labeling (TUNEL) staining and poly (ADP-ribose) polymerase (PARP) and annexin V western blotting. We correlated the neurodegeneration with sensorimotor deficits by conducting the animal behavioral tests including grid walk, balance beam, the inverted screen test, and the climb test. Our study demonstrated that the excess cell death or neurodegeneration correlated with the neuronal dysfunction and sensorimotor impairments associated with TBI.

## 1. Introduction

Traumatic brain injury (TBI) is defined as a debilitating effect on brain function resulting from a blunt or penetrating injury to the brain [1,2]. TBI is a major public health concern in the United States and it occurs predominantly due to automobile accidents, violence and falls. In a recent study, the investigators at the Centers of Disease Control addressed the scope and gravity of this ever-present problem. According to their study, in the year 2013 alone, a total of approximately 2.8 million TBI-related emergency department (ED) visits, hospitalizations, and deaths were recorded in the United States. The most vulnerable population was found to be elderly persons (≥75 years old), who sustained fall-related injuries or death [3].

Based on the Glasgow Coma Scale [4], TBI is classified into three categories—mild, moderate or severe—based on verbal responses, eye opening and motor responses of the patient. Mild TBI (mTBI) is the most common type of injury (80% of all cases) [5], and symptoms resolve within a few days or weeks. However, a recent study suggests that about 50% of individuals sustaining a single mTBI suffered long-term cognitive disability [6] indicating that half of the population with mTBI progresses to secondary neurodegeneration. Although the effects of the primary injury are immediate upon impact, secondary neurodegeneration is initiated by delayed, long-term cellular and biochemical changes at the site of impact that progressively lead to psychomotor dysfunction [7]. The mechanisms involved in secondary neurodegeneration in the aftermath of mTBI are not yet fully elucidated. However, oxidative stress has emerged as a central component of the process [8,9,10,11,12,13,14].

Recent studies from our lab and others have demonstrated that oxidative stress, Ca^2+^ influx, transforming growth factor-β (TGF-β) signaling and neuroinflammation are major mechanisms contributing to post-traumatic neurodegeneration [7,8,10,12,13,14,15,16]. Recently, we have found that reactive oxygen species (ROS) generated by the enzymatic action of nicotinamide adenine dinucleotide phosphate (NADPH) oxidase-1 (NOX1) leads to activation of matrix metalloproteinase-2 (MMP-2) that cleaves stromal cell-derived factor 1α (SDF-1α) to neurotoxic SDF-1 (5–67) fragment and ultimately leads to caspase-3 dependent apoptotic cell death [9]. In another recent study, we reported that the NOX1 activation causes upregulation of transforming growth factor-beta 1 (TGF-β1) that leads to caspase-3 mediated apoptosis via the phosphorylation of Smad2 and Smad3 proteins. Increased production of TGF-β1 exacerbates neuroinflammation via upregulation of inflammatory cytokines such as tumor necrosis factor-α (TNFα) and interleukin-1β (IL-1β) [13]. Additionally, calcium influx after TBI has been shown to activate caspase-1, which converts pro-IL-1β to IL-1β leading to inflammation and apoptotic cell death [10].

In the present study, we observed that the sensorimotor deficiencies in a mouse model of mild and moderate TBI was correlated with the neurodegeneration. Increasing secondary injury in mice resulted in a corresponding decrease in performance on all behavioral tests that targeted co-ordination, balance and muscle strength. Based on our previous findings, we investigated the possible involvement of caspase-3 dependent cell death pathway in the loss of sensorimotor function. During apoptosis, a cascade of signaling events leads to the activation of caspase-3, an enzyme that belongs to the family of cysteine proteases. Activated caspase-3 cleaves and inactivates poly (ADP-ribose) polymerase-1 (PARP-1) thus inhibiting DNA repair [17,18]. It simultaneously induces DNA fragmentation via the activation of caspase-dependent DNAse [19,20]. We also investigated the levels of Annexin V, a cellular protein known to be enriched in apoptotic cells [21]. The parameters of apoptotic cell death were found to be upregulated in mice subjected to mild and moderate TBI and were concomitant with the severity of the injury and loss of sensorimotor function.

## 2. Materials and Methods

### 2.1. Reagents

The primary antibody rabbit anti-cleaved caspase-3 (Cat. No. MAB835) was purchased from R and D systems (Minneapolis, MN, USA). Anti-cleaved PARP p85 (Cat. No. ab32064) was purchased from Abcam (Cambridge, MA, USA). Mouse anti-β-actin (Cat. No. PIMA515739) and rabbit anti-annexin V (Cat. No. PA5-27872) antibodies were purchased from Thermo Scientific (Rockford, IL, USA). All secondary Alexa Fluor-conjugated antibodies, TUNEL kit, and 4,6-diamidino-2-phenylindole (DAPI) were purchased from Invitrogen (Carlsbad, CA, USA).

### 2.2. Fluid Percussion Injury

Fluid percussion injury (FPI) was performed in adult male C57BL/6 mice (20–25 g, Taconic Biosciences Inc., Hudson, NY, USA). The animals used in this study were housed in John F Kennedy medical center animal facility care, fed ad libitum, and kept under a 12:12 light/dark cycle. Animals were maintained in sterile cages under pathogen-free conditions, and all procedures were approved by the Seton Hall University Institutional Animal Care and Use Committee (IACUC). All procedures were performed in accordance with the strict guidelines of the 8th edition of the Guide for the Care and Use of Laboratory Animals published by the US National Institutes of Health. We carried out surgeries for fluid percussion injury (FPI) during the 12 h light cycle. Standard surgical methods for lateral FPI were followed [9,10,13,22]. Briefly, 24 h before the injury, the mice were anesthetized with a ketamine/xylazine mixture (80 mg/kg ketamine and 10 mg/kg xylene), intra peritonial and surgically implanted with a Luer-Lok syringe hub to the right side of the skull in a stereotaxic device. This hub surrounds a craniotomy of the same size, positioned 3.0 mm posterior from and 3.5 mm lateral from the bregma. An additional cap that surrounds the syringe hub was applied. Two screws were implanted into the skull for additional support. Cranioplastic cement (AM Systems, Carlsborg, WA, USA) was applied around the syringe hub and between the cap to ensure fluid transmission and support. During surgery, body temperature (T_b_) was continuously monitored and maintained within normal ranges (36.5–37.5 °C) by a feedback temperature controller pad (model TC-1000; CWE, Ardmore, PA, USA). Prior to the injury, mice were anesthetized with 5% isoflurane until the foot-pinch reflex stopped. The animal was connected to a digitally controlled fluid percussion injury system-FP302 (AmScien Instruments, Richmond, VA, USA) and the injury was applied at 7–8 psi and 15–18 psi (6 animals each) with a pressure rise time of 8 ms. To avoid confusion, data figures were represented as 7 psi for mild injury and 15 psi for moderate injury. Mice exhibited apnea, loss of consciousness (LOC), and hyperextension of the tail and hind limbs after the injury. Similarly, control mice received the same anesthesia and surgery as the injured group and were connected to the dc-FPI and injury was not given. Six mice each were utilized in the injured and control groups for this study. Four animals per group are sufficient to get statistically significant results as per our previously reported studies and power analysis [9,12,13,23]. On 8th day after the injury, behavioral tests were conducted, and on 9th day, animals were anesthetized with ketamine/xylazine mixture and transcardially perfused with 1X PBS and 4% paraformaldehyde. Brain tissues were dissected out, embedded in an OCT (optimal cutting temperature) compound and kept frozen until analysis.

### 2.3. Western Blotting

Brain tissue samples (~50 mg) extracted below the injury from the neo-cortex were lysed with 500 mL of Cell Lytic-M buffer (Thermo Scientific, Rockford, IL, USA) containing a mixture of protease inhibitor (Sigma-Aldrich, St. Louis, MO, USA), and clear protein lysates were collected by centrifuging at 14,000 rcf for 10 min at 4 °C. The protein concentration in the tissue lysate was determined using the bicinchoninic acid (BCA) protein assay kit (Thermo Scientific, Rockford, IL, USA) [24]. Protein (20 μg) was resolved by 4–15% gradient SDS-PAGE gel (Biorad, Hercules, CA, USA). The blots were then transferred onto a nitrocellulose membrane, blocked with superblock (Thermo Scientific, Rockford, IL, USA), and incubated overnight with respective primary antibodies (1 mg/mL) at 4 °C. Subsequently, the membranes were then washed three times at 5-min intervals and incubated with horse-radish peroxidase-conjugated secondary antibodies (1:5000; Fisher Scientific, Rockford, IL, USA) for 1 h at room temperature (RT, approximately 25 °C). The membrane was rinsed three times with TBS-tween for 5 min at RT. Protein bands were detected using chemiluminescence western blot detection reagents (Abnova, Walnut, CA, USA) using Syngene gel documentation system (Frederick, MD, USA). Western blot bands were quantified as arbitrary densitometry intensity units using the ImageJ software package [14,25]. β-Actin was used as a loading control to normalize the quantification for cleaved caspase 3, Parp-p85 and annexin V.

### 2.4. Cell Death Analysis

Terminal deoxynucleotidyl transferase dUTP nick end-labeling (TUNEL, Invitrogen-Thermo Fisher Scientific, Carlsbad, CA, USA) assay kit was used to determine apoptosis in fixed tissue sections as per the manufacturer’s instructions [13,26]. To confirm the TUNEL assay, western blotting was carried out to determine the apoptosis using anti-cleaved poly-ADP-Ribose-Polymerase p85 (PARP p85) primary antibody (1 mg/mL; Promega, Madison, WI, USA). Anti-cleaved PARP p85 identifies the cleaved 85 kDa PARP fragment of 116 kDa poly-ADP-Ribose-Polymerase (PARP), which is an early marker for apoptosis and is mediated by a caspase-3 signaling pathway [27].

### 2.5. Behavioral Testing

To determine sensorimotor deficits, we evaluated four behavioral tests including grid walk, balance beam, inverted screen tests, and the climb test on 8th day after injury. All behavioral tests were performed within the 12 h light cycle, and experimenters were blinded to the treatment groups. In grid walk, mice were placed on an elevated metallic grid (45 × 100 cm) having 2.0 cm separation for each grid and allowed to walk for 2 min while being videotaped from below, as previously described [28,29]. Videos were later analyzed for total walking time and the number of forelimb foot errors for each foot. The grid walk errors were counted (four separate trials per test) and averaged from different trials. In balance beam, mice were tested for their ability to traverse a 100 cm-long in a 20-mm wide wooden dowel elevated 30 cm from the floor. In this test, latency to cross a flat beam and number of slips were recorded [30,31]. The videos were scored for ipsilateral and contralateral foot slips, and time required to reach the end of the beam. In the inverted screen test, we analyzed the combined forepaw and hind paw strength [32,33]. Mice were placed on a wire grid (2 × 2 cm), which is then inverted over a foam pad. The latency to fall was recorded. In the climb test, we used a 30-cm length metallic grid (2 × 2 cm). The grip was kept in a 60°-inclined position. The mouse was allowed to climb this grid and errors in walking/climbing or falling down time is recorded [30,34].

### 2.6. Statistical Analyses

Based on power analysis and previous studies [9,12,13,23], we estimated a sample size of *n* = 6 for detecting a significant effect of injury. No mortality was involved with the injury procedures and tissues samples from all animals were collected for analysis. Quantitative data are represented as mean ± SEM. All statistical tests were performed using the GraphPad Prism, V7 for Windows (GraphPad Software, Inc., San Diego, CA, USA) and data were analyzed with one-way ANOVA. Significant main effects or interactions were followed by *t*-tests with Bonferroni’s correction. A *p*-value < 0.05 was considered statistically significant.

## 3. Results

### 3.1. Mild and Moderate TBI Leads to Progression of Neurodegeneration

**Figure 1:**
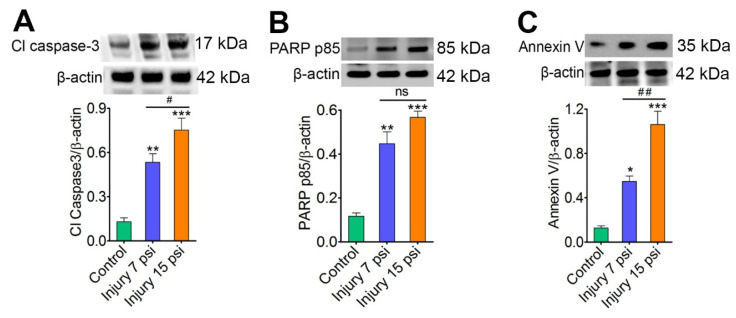
TBI induces apoptosis via caspase-3 in vivo: (**A–C**). Western blotting of cleaved caspase-3 (**A**), cleaved PARP (PARP p85) (**B**) and annexin V (**C**) in the brain cortex tissue lysates from uninjured control and FPI animals (7 psi and 15 psi) 9 days after injury. Values are mean ± SEM; *n* = 4. * *p* < 0.05, ** *p* < 0.01, *** *p* < 0.001 versus uninjured control; # *p* < 0.05; ## *p* < 0.01 versus 7 psi injury.

**Figure 2:**
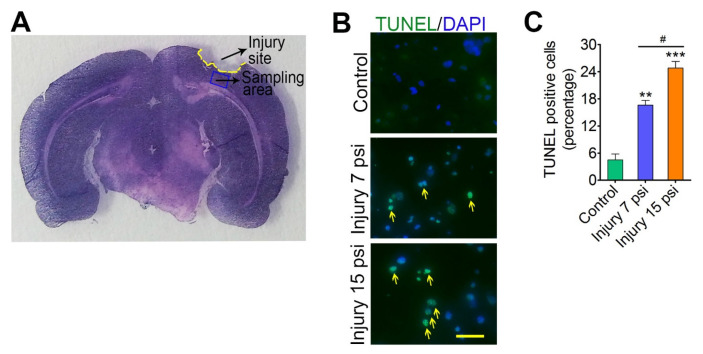
TUNEL staining (green) shows the apoptotic cells. (**A**) Hematoxylin and Eosin staining shows the FPI area (yellow mark) and the region evaluated (blue rectangular mark) in the coronary section of rat brain cortex; (**B**) TUNEL staining (green) shows the apoptotic cells in the mice subjected to control, 7 psi injury and 15 psi. Yellow arrows indicate TUNEL positive cells. Nucleus was counter-stained with DAPI (blue). Scale bar: 50 μm; (**C**) Percentage of TUNEL positive cells in mice brain slices 9 days after injury. Values are mean ± SEM; *n* = 4. ** *p* < 0.01, *** *p* < 0.001 versus uninjured control; # *p* < 0.05 versus 7 psi injury.

Neurodegeneration is one of the crucial events associated with secondary injury following TBI [9,13]. Here, we analyzed if mild and moderate FPI cause secondary neurodegeneration. To access neurodegeneration, we first analyzed the expression of the cleaved form of caspase-3 after mild (7 psi) and moderate (15 psi) fluid-percussion model of traumatic brain injury (TBI) in mice. Immunoblot analysis identified a significant increase in cleaved forms of caspases-3 in both mild and moderate TBI when compared to control animals (*p* < 0.05; *p* < 0.001) (Figure 1A). We next assessed the level of cleaved PARP fragment, a marker of apoptosis. Western blot analysis using anti-PARP p85 antibody showed a significant increase in the level of the cleaved fragment (85 kDa PARP fragment) in both 7 psi (*p* < 0.001, *n* = 4) and 15 psi (*p* < 0.001, *n* = 4) FPI groups as compared to control groups. The expression level of cleaved PARP fragment in 15 psi FPI group was also significantly higher than 7 psi group (*p* < 0.05) (Figure 1B). A similar significant increase in the expression of annexin V was observed in both 7 psi and 15 psi groups as compared to the control group (Figure 1C). We further investigated the mice brain cortex tissue sections (sampling area shows in Figure 2A) from control and 7 psi and 15 psi FPI animals for TUNEL assay. Analysis of TUNEL positive cells showed numerous apoptotic cells in cortical tissue 8 days after injury (*p* < 0.001, *n* = 4) (Figure 2B,C). Taken together, the results clearly suggest that neurodegeneration is prevalent in FPI injury in mice and the injury is more pronounced in moderate FPI than mild.

### 3.2. Mild and Moderate TBI Causes Sensorimotor Deficits Concomitant with Neurodegeneration

**Figure 3:**
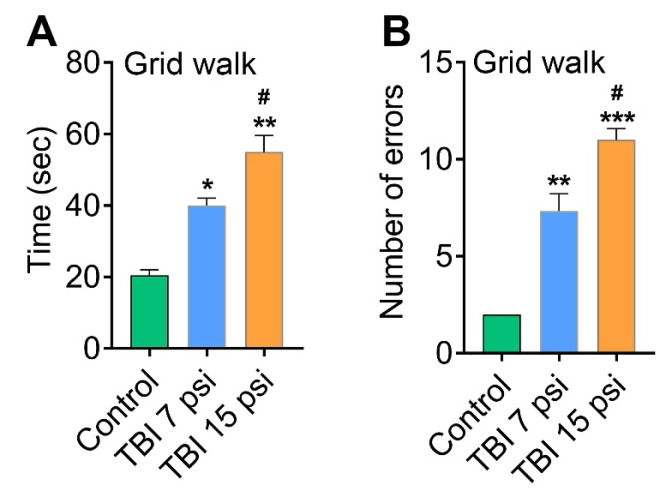
TBI causes sensorimotor deficits. Behavioral tests conducted at 8 DPI to analyze the effect of mild and moderate TBI on sensory and motor function. (**A**,**B**) Grid-walk analysis demonstrated that both 7 and 15 psi injured mice made more time to finish the grid walk (**A**) with more number of errors (**B**) than control animals. Values are mean ± SEM; *n* = 6. * *p* < 0.05, ** *p* < 0.01, *** *p* < 0.001 versus uninjured control; # *p* < 0.05 versus 7 psi injury.

**Figure 4:**
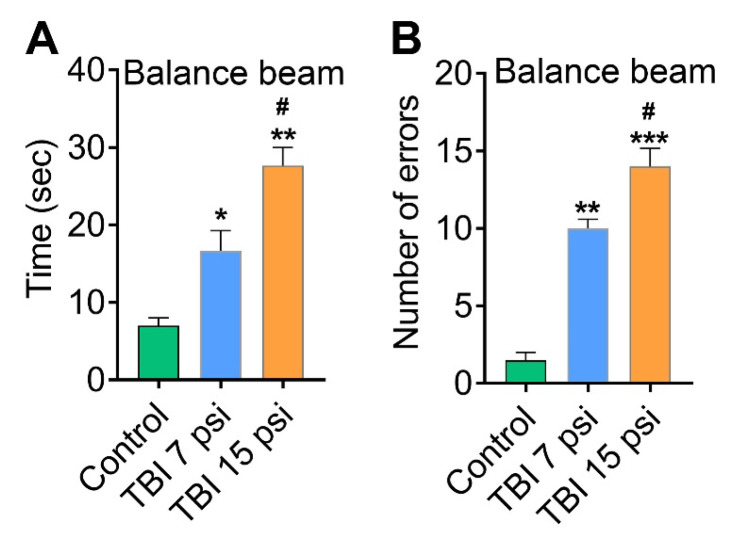
Balance beam test: (**A,B**). Balance beam test was performed on the same animals and both 7 and 15 psi injured mice displayed more time spent (**A**) in the balance beam with more number of errors (**B**) than control mice. Values are mean ± SEM; *n* = 6. * *p* < 0.05, ** *p* < 0.01, *** *p* < 0.001 versus uninjured control; # *p* < 0.05 versus 7 psi injury.

To validate whether sensory and motor deficit associated with mild and moderate TBI correlates to neurodegeneration, a cohort of behavioral tests was performed. The effect of mild (7 psi) and moderate (15 psi) TBI on functional sensory and motor deficit was determined with four behavioral tests: grid walk, balance beam, the inverted screen test and the climbing test. Results from the grid walk test showed that both 7 and 15 psi injury mice 8 days after injury had a significant latency (*n* = 6) (Figure 3A) and more number of slips (errors) (*n* = 6) (Figure 3B) were observed as compared to control animals. Similarly, the balance beam test for 7 and 15 psi injury mice showed a significant difference in latency and number of slips from each other and when compared to the control mice (Figure 4A,B). Among the mild (7 psi) and moderate (15psi) groups, 15 psi FPI showed maximum latency and number of errors and were statistically significant as compared to 7 psi group (*p* < 0.05).

**Figure 5:**
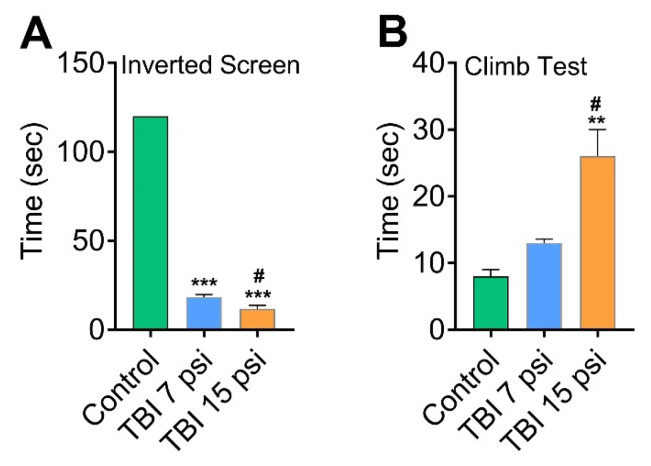
FPI causes deficits in motor skills and neuromuscular strength: (**A,B**). Inverted screen (**A**) and climb test (**B**) to access motor skills and neuromuscular strength demonstrated deficit in inverted screen test and climb test. Values are mean ± SEM; *n* = 6. ** *p* < 0.01, *** *p* < 0.001 versus uninjured control; # *p* < 0.05 versus 7 psi injury.

Next, we used the inverted screen and climbing tests to measure motor skills and neuromuscular strength and further understand the skeletal phenotype associated with mild and moderate FPI in mice model. We used the inverted screen and climb tests over 120 s to assess the duration to fall and the duration to climb over this period. When comparing the performance of independent cohorts of control, 7 psi and 15 psi mice on time spent gripping the inverted screen, both 7 psi (*p* < 0.05 versus control, *n* = 6) and 15 psi (*p* < 0.05 versus control, *n* = 6) groups show significant differences as compared to the control group. However, time suspended on the inverted screen progressively worsens with moderate (15 psi) FPI (*p* < 0.05 versus 7 psi) (Figure 5A). In the climb test, a decrease in neuromuscular strength (increased duration to climb) was observed in 15 psi injury group as compared to the control animals (*p* < 0.05 versus control) but no significant difference was observed between 7 psi injury group and the control group (*p* > 0.05, *n* = 6) (Figure 5B). Taken together, these results clearly indicate the link between neurodegeneration and sensorimotor deficits associated with TBI.

## 4. Discussion

Here, we tested the hypothesis that neurodegeneration caused by TBI leads to impairments in sensorimotor function. The results from this study show that TBI mimicked by mild and moderate fluid percussion injury (FPI) in mice causes deterioration in sensorimotor function and the extent of sensorimotor deficits correlates to neurodegeneration as marked by activation of the apoptotic signaling pathway.

Traumatic brain injury is frequently associated with secondary neurodegeneration that leads to impairment of sensory motor functions [35,36,37]. Our result agrees with previous studies that demonstrate progressive brain atrophy and sensorimotor deficits in rodent models of traumatic brain injury [38,39,40,41]. The parietal lobes and their related circuitry are particularly more vulnerable to traumatic damage; hence executive dysfunction is prevalent [42,43]. Thus, in our study, we specifically analyzed the parietal lobe to assess if neurodegeneration associated with TBI correlates to sensorimotor deficits. In our study, injury severity was assessed with a battery of sensorimotor behavioral tasks to measure functional sensorimotor deficits following FPI. In the brain samples of mice sacrificed on 9th day post injury, both mild and moderate FPI showed a significant increase in the markers of apoptosis including cleaved caspase-3, and cleaved PARP and annexin V when compared to uninjured control animals in the ipsilateral cortices. Moreover, our data supports the hypothesis that the extent of injury exacerbates from mild to moderate FPI and is concomitant with the sensorimotor deficits. 

In our study, neurodegeneration was monitored 9 days post injury since previous studies demonstrated that TBI patients earlier in their course of injury (within 9 days) continue to exhibit ongoing neurodegeneration [44]. This is in accordance with other studies that validate progression of neurodegeneration that continues years post injury [45]. Previous studies demonstrate that cognitive impairments normalize by 5 dpi. However, persistent abnormalities were observed with advanced magnetic resonance imaging (MRI) and blood proteomics, suggesting traditional diffusion-tensor measures as a less sensitive outcome to access neurological deficits [40]. By contrast, another study demonstrates that sensorimotor deficits remain prevalent even after 12 weeks of FPI despite increased fractional anisotropy, axial diffusivity, and tract density. This point, towards long-term recovery, is evident. However, it does not remediate all behavioral deficits [39]. Thus, in our studies, neurodegeneration was assessed 9 dpi to assess behavioral deficits associated with lateral fluid percussion injury.

Our approach used the open-skull FPI model to study TBI-induced neurodegeneration and sensorimotor deficits. The clinical validity of the open-skull approach is of concern since the majority of clinical TBI is closed head injury and only 0.8 to 3% of patients experience open head injury [46,47], thus compromising monitoring of intracranial pressure typical of TBI. However, despite the limitations of lateral FPI, numerous studies have demonstrated that the FPI model mimics an extensive range of pathological features associated with human TBI thus rendering it as a relevant clinical model of human TBI [48]. As the magnitude of injury can be controlled through this approach, neurodegeneration and neurological deficits associated with mild and moderate TBI can efficiently be studied.

Apnea associated with TBI could also lead to neuropathology and contributes to the progression of injury. Previous studies demonstrated that fluid percussion injury could produce either irreversible apnea and death or transient apnea [49,50,51]. In our study, we observed transient apnea following both mild and moderate injury. However, no mortality rate was evident following apnea. The duration of apnea persists for approximately 10–15 s followed by restoration of regular respiration and all animals undergoing transient apnea survived 9 dpi. Thus, transient apnea was probably not contributing to neuropathology to animals that survived.

Taken together, our data support that secondary injury exacerbates sensorimotor deficits after TBI and directly compliment previously reported experimental brain-injury studies [38,39]. We interpret these findings to indicate that neurodegeneration following FPI exacerbates sensorimotor deficits and thus provides direct evidence that incidence of neurodegeneration after FPI may potentially result in increased neurological deficits and thereby warrant strict monitoring of these events.
